# Effect of drought stress on natural rubber biosynthesis and quality in *Taraxacum kok-saghyz* roots

**DOI:** 10.1371/journal.pone.0295694

**Published:** 2024-01-22

**Authors:** Seyed Shahab Hedayat Mofidi, Mohammad Reza Naghavi, Manijeh Sabokdast, Parisa Jariani, Meisam Zargar, Katrina Cornish

**Affiliations:** 1 Division of Biotechnology, Department of Agronomy and Plant Breeding, College of Agricultural and Natural Resources, University of Tehran, Karaj, Iran; 2 Department of Agrobiotechnology, Institute of Agriculture, RUDN University, Moscow, Russia; 3 Departments of Horticulture and Crop Science, and Food, Agricultural and Biological Engineering, The Ohio State University, Wooster, OH, United States of America; Hainan University, CHINA

## Abstract

*Taraxacum kok-saghyz* (TKS) is a potential source of natural rubber (NR) that can be grown in temperate regions with limited water availability. However, the effect of drought stress on NR production and properties in TKS isn’t well studied. This study examined how different levels of drought stress (30, 60 and 90%) influenced the NR content, molecular weight (Mw), glass transition temperature (Tg), gene expression, and biochemical parameters in TKS roots. The results showed that drought stress didn’t significantly change the NR content, but increased the Mw and the expression of *CPT* and *SRPP* genes, which are involved in NR biosynthesis. The NR from TKS roots (TNR) had a high Mw of 994,000 g/mol and a low Tg of below -60°C under normal irrigation, indicating its suitability for industrial applications. Drought stress also triggered the accumulation of proline, H_2_O_2_, MDA, and antioxidant enzymes (CAT, APX, GPX) in TKS roots significantly, indicating a drought tolerance mechanism. These findings suggest that TKS can produce high-quality NR under drought stress conditions and provide a sustainable alternative to conventional NR sources.

## 1. Introduction

Natural polyisoprenes are divided into two major groups namely, *cis* and *trans*- polyisoprene. Natural rubber (NR) is *cis*-1,4-polyisoprene, and is one of the most valuable biopolymers in the world and is used as a critical raw material in numerous products [1]. It is energetically expensive to biosynthesize, a terminal carbon sink, and is a secondary metabolite in plant cells. Although it is made in at least 2500 plant species its function in plants is poorly understood and disparate among species [1, 2]. The tropical para rubber tree (*Hevea brasiliensis*) cultivated predominately in southeast Asia is the only significant commercial source of NR production in the world [3]. The NR supply chain is insecure due to growing demand, price volatility, increasing worker costs, commerce policy, rain forest destruction moratoria, and susceptibility to many diseases [4]. Biological and geographical diversity is needed. Among rubber-producing species known to synthesize high molecular weight rubber, *Parthenium argentatum* (also called guayule), and *Taraxacum kok-saghyz* (also known as rubber dandelion, TK, TKS) have received growing attention in the past few years, as potential commercial sources of NR [5–7]. These species provide a sustainable and renewable source of NR that offers an alternative to Hevea rubber in semi-arid temperate regions, respectively, hence reducing dependency tropical rubber [8]. Guayule rubber is especially attractive as it is hypoallergenic, unlike normal Hevea rubber which can trigger allergic reactions in some individuals [9]. TKS is an annual herbaceous plant, originating in Kazakhstan and neighboring countries, widely adapted to temperate regions, that makes rubber in its root laticifers similar in quality to NR from Hevea [6, 10, 11].

Drought stress is a major global challenge that affects the productivity and quality of crops. Drought triggers physiological alterations and metabolic impairment such as lipid membrane peroxidation, protein folding issues, metabolite degradation [12] accumulation of reactive oxygen species (ROS) and respiratory metabolism inhibition, which may result from a reduction in antioxidants. To scavenge reactive oxygen, antioxidant enzymes are vital. Some of the enzymes that protect plants from oxidative stress are CAT, which breaks down hydrogen peroxide, APX, which reduces ascorbic acid, and GPX, which uses guaiacol as a substrate [13]. Environmental factors such as low temperature and water scarcity may trigger the production of rubber in some plants [14]. A key ingredient for making rubber is isopentenyl pyrophosphate (IPP), which can be produced through two distinct routes: the pathway of mevalonic acid (MVA) and the pathway of methylerythritol phosphate (MEP) [15–17]. The membrane-bound enzyme complex responsible for the synthesis of the NR polymer (rubber transferase, RT-ase) is not yet fully understood [1].

This study examined the expression levels of four key genes likely related to rubber biosynthesis in response to drought. These genes are *SRPP* (which binds to small rubber particles), *CPT1* (which catalyzes the formation of *cis*-prenyl chains) *HMGR* (hydroxy methylglutaryl coenzyme A reductase) and *HMGS* (hydroxy methylglutaryl coenzyme A synthase). The *SRPP* gene was first recognized as encoding a protein associated with rubber particles in *Hevea brasiliensis*. Over-expression of *SRPP3* increased polyisoprene content in TKS roots [18] (Schmidt et al., 2010), whilst *SRPP3* RNA interference (RNAi) caused a significant decrease in rubber content and rubber molecular weight [19]. *SRPP* is also thought to play a role in rubber particle stability. *CPT* (*cis*-prenyltransferase) associated with rubber particles is a key catalytic enzyme involved in the polymerization reaction [20]. Several studies have reported on the genes and pathways involved in TKS drought stress response and tolerance. For example, Cheng et al. [18] identified 72 WRKY transcription factors (TFs) in TKS and analyzed their expression patterns under various abiotic stresses and hormonal treatments. They found that some *TkWRKY* genes were significantly upregulated or downregulated by drought stress, suggesting their roles in regulating TKS drought response. WRKY TFs are known to play crucial roles in plant physiological processes and stress responses by modulating the expression of downstream genes [21]. Another study compared the transcriptome profiles of drought-inducible genes in Arabidopsis and rice using microarrays. They identified 79 common drought-inducible genes in both species, which were mainly involved in signal transduction, transcription, protein modification, and metabolism. They also found that some drought-inducible genes were specific to each species, reflecting their different adaptation strategies to drought stress. They suggested that the common drought-inducible genes could be used as candidate genes for improving drought tolerance in various crops, including TKS [22]. A study reported on the genome of a drought-tolerant plant, Resurrection plant (*Selaginella lepidophylla*). They showed that many genes are involved in keeping plants from withering, remaining healthy and resistant to a lack of oxygen. They also compared the genome of this plant with other plant genomes and found some unique features that could help to understand the evolution of plant drought tolerance [23]. The objective of this study was to investigate the effects of drought stress on the expression of genes involved in NR biosynthesis and the NR content in the roots of TKS plants. This is the first and novel study to explore the impact and mechanisms of drought stress on NR biosynthesis and quality in TKS, an alternative source of NR with potential for cultivation and utilization. Previous studies on TKS have mainly focused on its agronomic traits, genetic diversity, and rubber biosynthesis genes [24]. However, there is a lack of information on how TKS responds to drought stress and how it affects its rubber production and quality. Therefore, this study fills a gap in the literature by investigating the effects of drought stress on the NR biosynthesis and quality in TKS.

## 2. Materials and methods

### 2.1. Experimental design and drought stress treatments

TKS seeds of accession number W6 35164 were acquired from the USDA-ARS (Regional Plant Introduction Station, Washington State, U.S.A.). Germination rates were improved to above 80%, by first soaking the seeds in water for 20 h, followed by 2% KNO_3_ for 4 h. Each seed was rinsed twice with distilled water and sown in a growth tray that had drain holes and a mixture of perlite, cocopeat and peat moss in equal parts. Thereafter, when plants reached the six-leaf stage, they were transferred to pots containing clay loam mixed with gravel (2 mm to 5 mm) at a ratio of 3:1, about 24% sand and the soil content include sand (0.05 mm to 2 mm), 45% silt (0.002 mm to 0.05 mm) and clay 31% clay (< 0.002mm). Potted plants were grown in the research greenhouse of the College of Agricultural and Natural Resources, University of Tehran, Karaj, at 35° N and 50° E, with 25°C Day and 16°C night temperatures and a 16 h day and 8 h dark photoperiod. The plants were watered and fertilized (N_20_P_20_K_20_) about four months until 50% of plants reached sexual reproduction. Additional seedlings were planted in the field with clay loam soil.

#### 2.1.1. Drought stress treatment in the greenhouse

**Fig 1:**
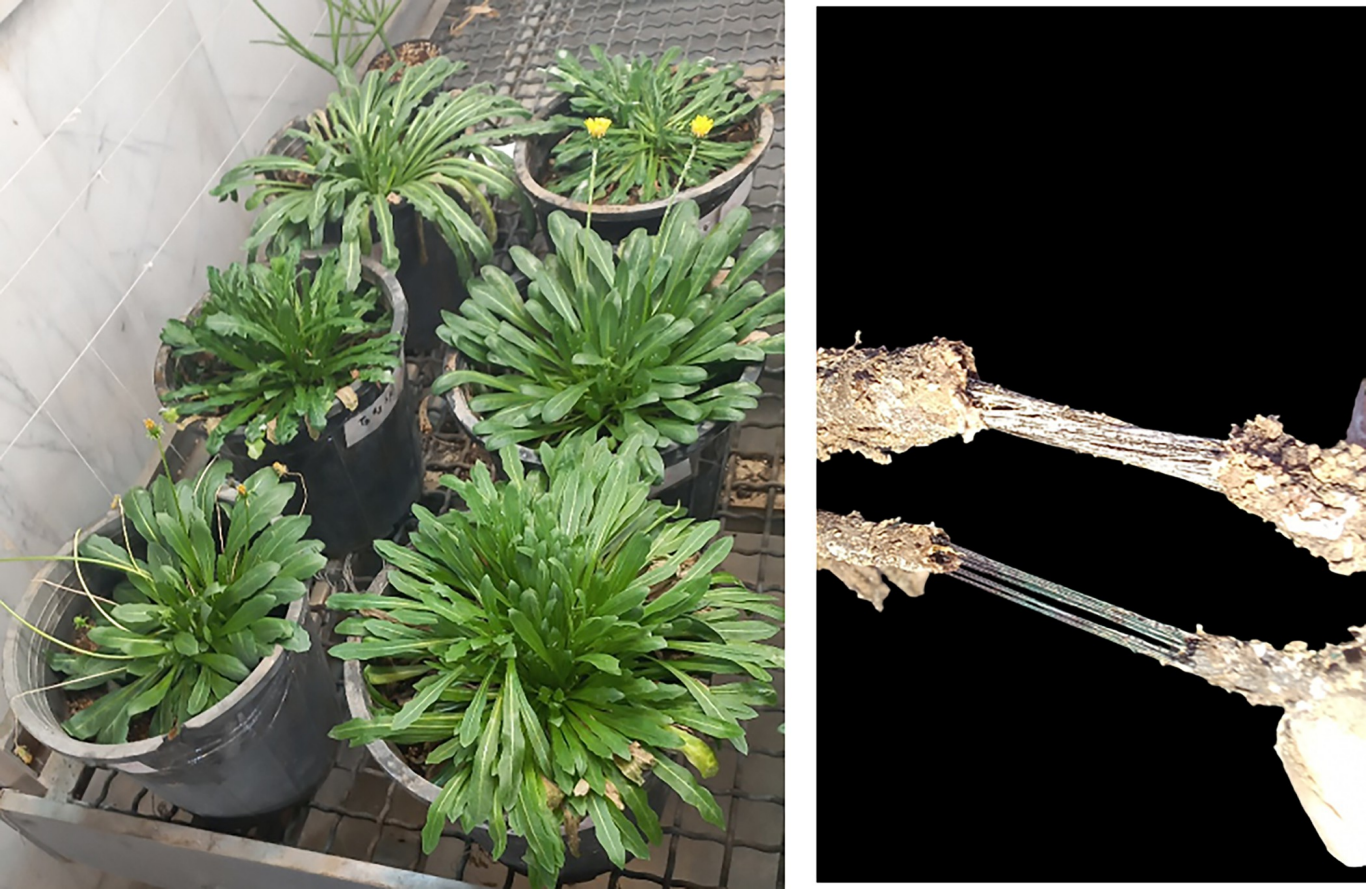
(**a**); *T*. *kok-saghyz* plants and (**b**); Natural rubber (TNR) strands stretched between broken root sections.

The experiment followed a Randomized Complete Block Design (RCBD) with three blocks. Each block had six plants in it. When 50% of the plants began flowering, plants were exposed to the following treatments at three levels of water availability: 30% of field capacity (FC) (severe stress), 60% of FC (mild stress) and 90% of FC (sufficient-irrigation treatment) (Fig 1A). After 21 days, plants were taken out of the pots and the samples were collected simultaneously from both tissue of root tips (two cm in length) and leaves of TKS, with a total of 15 leaves and the samples were rapidly frozen in liquid nitrogen and then kept at -80°C for analysis.

#### 2.1.2. Drought stress treatment in the field

Soil moisture measurements were used to guide irrigation treatments according to the percentage of maximum allowable depletion (MAD) of the total available water of the soil [25]. Drought stress treatments were based on 10, 40 and 70% MAD of total available soil water, representing the control, mild and severe drought stress, respectively. The amount of soil water content was estimated using a TDR probe (TDR Model 100) system.

### 2.2. Physiological measurements

#### 2.2.1. Leaf extract preparation

Leaf samples (0.2 g) were chopped and powdered in liquid nitrogen using a mortar and pestle then moved to tubes with a capacity of 2 mL. Thereafter, 1 mL of extraction buffer consisting of (Tris-HCl(C_4_H_11_NO_3_HCl) (Merck, Germany), 1 M, pH 6.8 and 2% polyvinyl pyrrolidone (PVP) (Merck, Germany) was added and mixed well. The extract underwent centrifugation at 4°C with a force of 22,500 g for half an hour. The liquid fractions were separated and kept at -20°C for later enzyme activity assays [26–28].

#### 2.2.2. Evaluation of malondialdehyde (MDA) content

The Peever and Higgins method based on the thiobarbituric acid (TBA) reaction was used to measure the level of malondialdehyde (MDA), a marker of lipid peroxidation. First, 0.1 g of fresh TKS leaves were homogenized in 5 mL of 0.1% (w/v) trichloroacetic acid (TCA) and the homogenate was centrifuged at 10,000 g for 5 min. Then, 1 mL of the supernatant was mixed with 4 mL of 20% TCA containing 0.5% (w/v) TBA and the mixture was heated at 95°C for 30 min. After being cooled on ice, the mixture was centrifuged again at 10,000 g for 15 min and the absorbance of the supernatant was measured at 532 and 600 nm [29].

#### 2.2.3. Measurement of proline level

To measure the free proline in the fresh leaves of TKS under different treatments, the acid ninhydrin method was applied. A 0.5 g sample of fresh leaves was homogenized with 10 mL of 3% sulfosalicylic acid on ice. The homogenates were centrifuged at 22,500 g for 15 min at 4°C and filtered through filter paper. The filtrates were collected in 15 mL Falcon tubes and mixed with 2 mL of acid ninhydrin and 2 mL of glacial acetic acid. The mixture was incubated in a water bath at 65°C for 1 h and then cooled on ice. After adding 4 mL of toluene to each tube and vortexing for 20 sec, the absorbance of the toluene layer was measured at 520 nm using a plate reader device (EON Biotek, Highland Park, Winooski, Vermont, USA). The proline concentration of the samples was calculated from the absorbance values at 520 nm using a standard curve of known proline concentrations. The proline content was then expressed as micrograms of proline per gram of dry weight (μg/g DW) using the following formula:

$M=\frac{C\times V}{W}$, where M is the proline content in μg/g DW, C is the proline concentration of the standard solution in μg/mL, V is the volume of the supernatant in mL, and W is the weight of the fresh leaves in g [30, 31].

#### 2.2.4.Evaluation of H_2_O_2_ content

To measure the H_2_O_2_ content in fresh leaves, the method of [32] was followed. A 0.5 g sample of leaf tissue was ground in 2.5 mL of 1% TCA solution and spun at 15,000 g for 15 min. Then, 0.25 mL of the clear liquid was combined with 0.25 mL of 10 mM potassium phosphate buffer (pH 7) and 0.5 mL of 1M potassium iodide solution. The mixture’s absorbance at 390 nm was read by a plate reader and matched to a standard curve of H_2_O_2_ solutions ranging from 2 to 10 mM.

#### 2.2.5. Determination of catalase (CAT) content

Catalase activity was measured by adding 10 μl of leaf extract (prepared as described in section 2.2.1) to 1,990 μl of reaction buffer containing 50 mM potassium phosphate buffer and 50 mM H_2_O_2_. The decrease in absorbance at 240 nm due to the decomposition of H_2_O_2_ by catalase was monitored using a spectrophotometer [33, 34].

#### 2.2.6. Determination of ascorbate peroxidase (APX)

Ascorbate peroxidase (APX) activity was measured following the method of Nakano and Asada, [35]. The assay mixture contained 50 mM potassium phosphate buffer, 0.5 mM ascorbic acid and 1 mM H_2_O_2_. A 10 μl aliquot of leaf extract was added to 1990 μl of the assay mixture and the decrease in absorbance at 290 nm due to the oxidation of ascorbic acid by APX was recorded spectrophotometrically. The activity was expressed as ΔA290 min^−1^mg^−1^ protein.

#### 2.2.7. Determination of guaiacol peroxidase (GPX) content

The method of Chance and Maehly, [36] was followed to assay guaiacol peroxidase (GPX) activity. The reaction buffer was composed of 25 mL potassium phosphate (10 mM), 25 mL H_2_O_2_ and 25 mL guaiacol. Leaf extract (10 μL) was added to 1,990 μL of this reaction buffer, mixed well and the GPX activity was spectrophotometrically determined at 470 nm.

#### 2.2.8 Cluster analysis

To examine the patterns of similarity among the physiological parameters and the drought stress treatments, cluster analysis was performed using the R package. A heatmap was generated to visualize the clustering results based on the Euclidean distance method. The hierarchical clustering of the physiological parameters and the drought stress treatments are shown by the dendrograms on the top and left sides of the heatmap, respectively. The heatmap indicates the degree of association between the physiological parameters and the drought stress treatments by using a color scale [37, 38].

### 2.3. RNA isolation and reverse transcription

Following the MIQE guidelines [39], real-time PCR was conducted. Root tip tissue (100 mg) from TKS was collected and the sample was pulverized into a fine powder using liquid nitrogen and a cold mortar and pestle. The improved CTAB method [40] was used to isolate and purify total RNA. A 1% agarose gel and Nanodrop ND-1000 were used to check the quality and quantity of total RNA, respectively. To remove any possible genomic DNA contamination (confirmed by PCR), the total RNA was digested with DNase I, RNase-Free DNase Set (Fermentas, Waltham, MA). Then, using a cDNA synthesis kit (RP1300 Reverse Transcription Kit made by SMOBIO Taiwan), the first strand cDNA was generated from the total RNA following the protocol provided by the manufacturer.

### 2.4. Primer design and quantitative real-time PCR (qRT-PCR)

**Fig 2:**
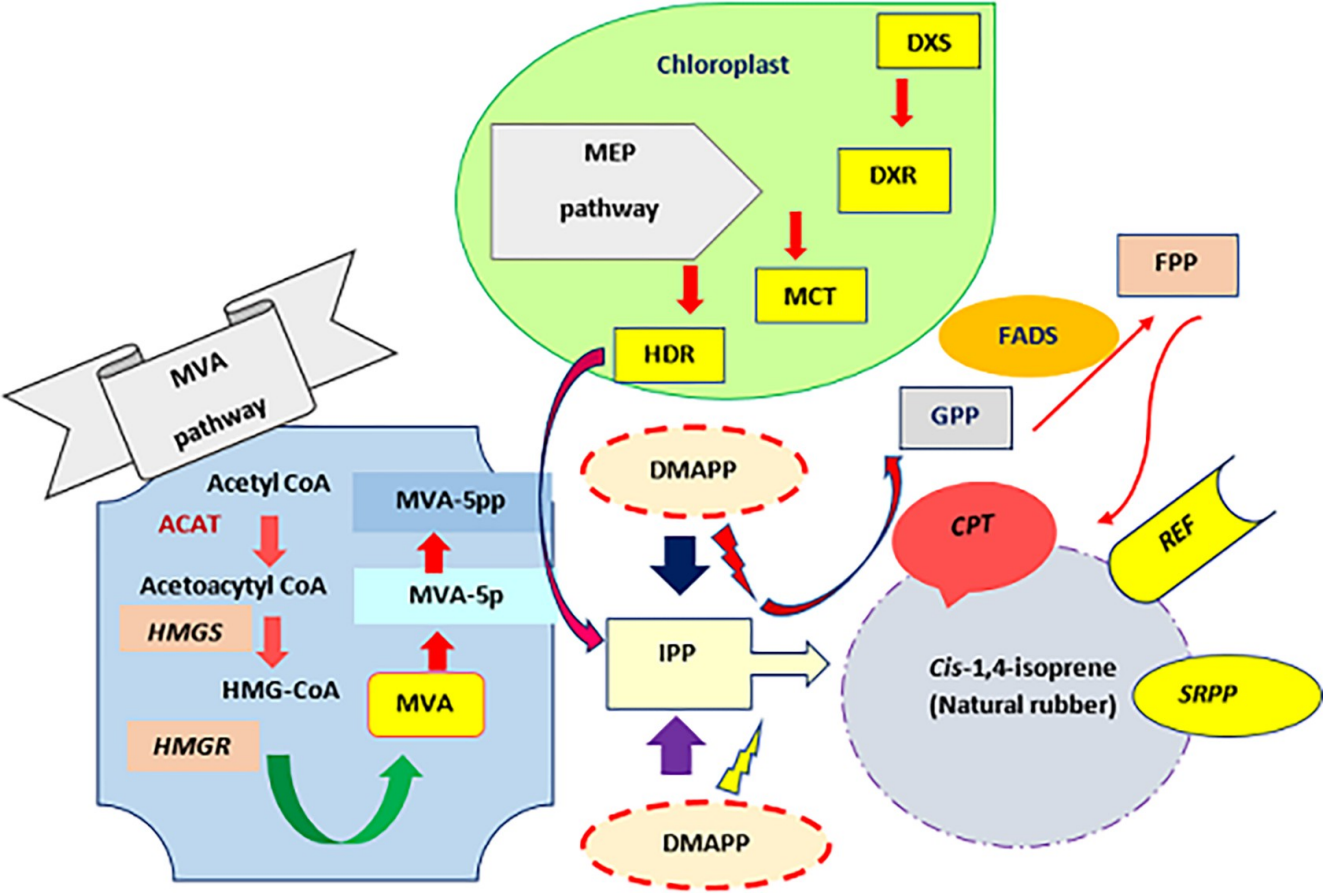
Natural rubber biosynthesis pathway in plants. The monomer isopentenyl pyrophosphate (IPP) is derived from two sources: the mevalonic acid (MVA) pathway and the methylerythritol (MEP) pathway. IPP is converted to dimethylallyl pyrophosphate (DMAPP) by IPP-isomerase. DMAPP and IPP are then polymerized by cis-prenyltransferase (CPT) to form *cis*-1,4-polyisoprene, the main component of NR. The polymerization occurs on the surface of small rubber particles, which are coated with small rubber particle protein (SRPP). Some key enzymes of the MVA pathway are hydroxy methylglutaryl coenzyme A synthase (HMGS) and hydroxy methylglutaryl coenzyme A reductase (HMGR).

The corresponding primers of the selected genes (Fig 2), *CPT1* (KM401657.1), *HMGR3* (KT899404.1), *HMGS1* (KT899404.1) and *SRPP2* (KT899433.1), were designed using Oligo7 primer analysis software. Primers were checked with the Oligo-analyzer and NCBI/Primer-BLAST web tools software. Table 1 shows the forward and reverse primer sequences used for qRT-PCR. Real time PCR was performed for 40 cycles with SYBR green Master Mix 2X in a Rotor-gene 6000 series software (QIAGEN’s real-time PCR system) following the manufacturer’s instructions. Each cycle consisted of three steps: 95°C for 15 sec, 59–65°C (based on the annealing temperature from PCR gradient results) for 20 sec, and 72°C for 20 sec. The relative expression of the different genes was then calculated. All the reactions were repeated with three technical replicates for statistical analysis and relative gene expression was calculated using REST software (REST 2009 V2.0.13).

**Table 1:** Specific primers used in qRT-PCR.

Target Genes	Gene bank accession number	Primer sequences (Sequence in 5′-3′ direction)
*CPT1*	KM401657.1	F: GTGTCATAGCTTCTCGCCCAA R: ATGGTGACGTACTTAACTCCGAT
*HMGS1*	KT899404.1	F: CCATAGGACTCGCACAAGATTGC R: CGATTACCGTTTCACTCCCGACTTC
*SRPP2*	KT899433.1	F: TTTGCTGATAATAAGGTTGCCCA R: CTTTTACGGTTTCCACTACACCA
*HMGR3* *Actin*	KT899404.1 KT965025.1	F: CCGTTTTCAACAAATCAAGCCGAT R: ACCATGTTCATCCCCATTGCATC F: GTATCCATGAGACCACCTACAAC R: GTCAGCAATACCAGGGAACATA

### 2.5. TKS root tissue preparation for rubber extraction

TKS roots from three different irrigation regimes were harvested from greenhouse-grown TKS plants. The plants were removed from their pots, thoroughly washed with clean water, dried at room temperature for about 1 h and finally aerial organs of plant was separated from the crown of plant by a cutter. The roots were sliced and oven-dried at 50°C for 4–7 days (Fig 1B). Dried roots were cut into 2 mm pieces. Finally, the chopped dried roots were kept in a zipped bag at 4°C in the dark until TNR extraction [41].

### 2.6. Extraction and characterization of TNR

**Fig 3:**
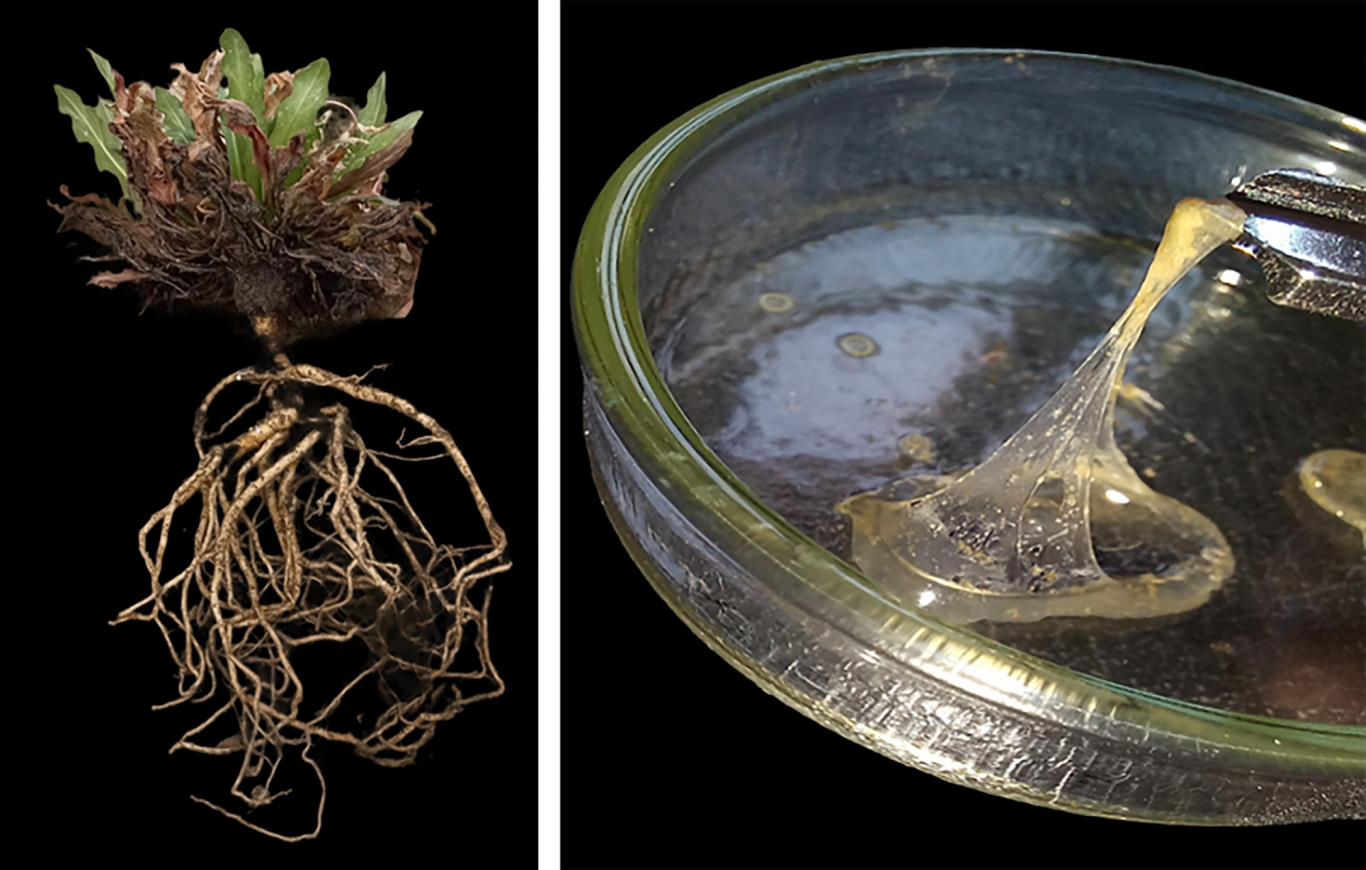
Plant (left) and dried TNR in a petri dish (right).

TNR was extracted using sequential solvent extraction with acetone and hexane [42] Roots harvested from both the greenhouse and the field with a weight of approximately 0.1 g per sample were stirred for 24 h in acetone (20 mL acetone/g ground root), then centrifuged at 2,800 g for 10 min. After decanting the supernatant, the pellet was washed with acetone two times. Each pellet was then mixed in hexane for 6 h (20 mL hexane/g ground root). The slurry was centrifuged at 2,800 g for 10 min. Each supernatant was transferred to a pre-weighed glass petri dish and dried in a 50°C oven for 2 h (Fig 3) until the solvent had completely evaporated. The extracted TNR was weighed. TNR samples were left in the petri dishes, covered and sealed in plastic, and the samples were kept in a light-free environment at 4°C until further analysis.

#### 2.6.1. FTIR analysis of NR

15 to 20 mg of extracted of rubber were stretched on KBr cells [43, 44] and analyzed by Fourier transform infrared (FTIR) spectroscopy using an ALPHA II, Brucker device.

#### 2.6.2. GPC analysis of NR

The molecular weight and polydispersity of extracted TNR was assessed through gel permeation chromatography (GPC). TNR (3 mg) was dissolved overnight in tetrahydrofuran (THF) at 25°C with moderate shaking. The solutions were filtered through a PTEF filter (0.45 μM) and injected (100 μl) into a GPC (Agilent 1100 series, Agilent Technologies, Palo Alto, CA, USA) with a RI detector and two PLgel mixed-C columns (10 μM, 300 mm × 7.5 mm) in series. The flow rate and the temperature were 1 mL/min and 35°C, respectively. Polystyrene standards (ranging from 580 to 19,560,0000 molar mass) were used to estimate molecular weights [41, 45, 46].

#### 2.6.3. DSC analysis of NR

The thermal responses of TNR samples (10 mg) to heat were analyzed by differential scanning calorimetry (DSC) (Perkin-Elmer DCS model 8000) under a nitrogen atmosphere. To erase their thermal memory, the rubber samples were kept at 30°C for 10 min. Then they were warmed up from -80°C to 180°C at a speed of 10°C/min [47].

### 2.7. Statistical analysis

The results of physiological parameter measurements and total rubber content were analyzed using SAS (9.4) software. The least significant difference (LSD) test was used to compare the means of data at (*p* ≤ 0.05 and *p* ≤ 0.01) significance levels. The 2^-ΔΔCT^ method [48] was applied to statistically analyses the relative expression levels of each selected gene with three technical replicates.

## 3. Results

### 3.1. Physiological measurements

**Fig 4:**
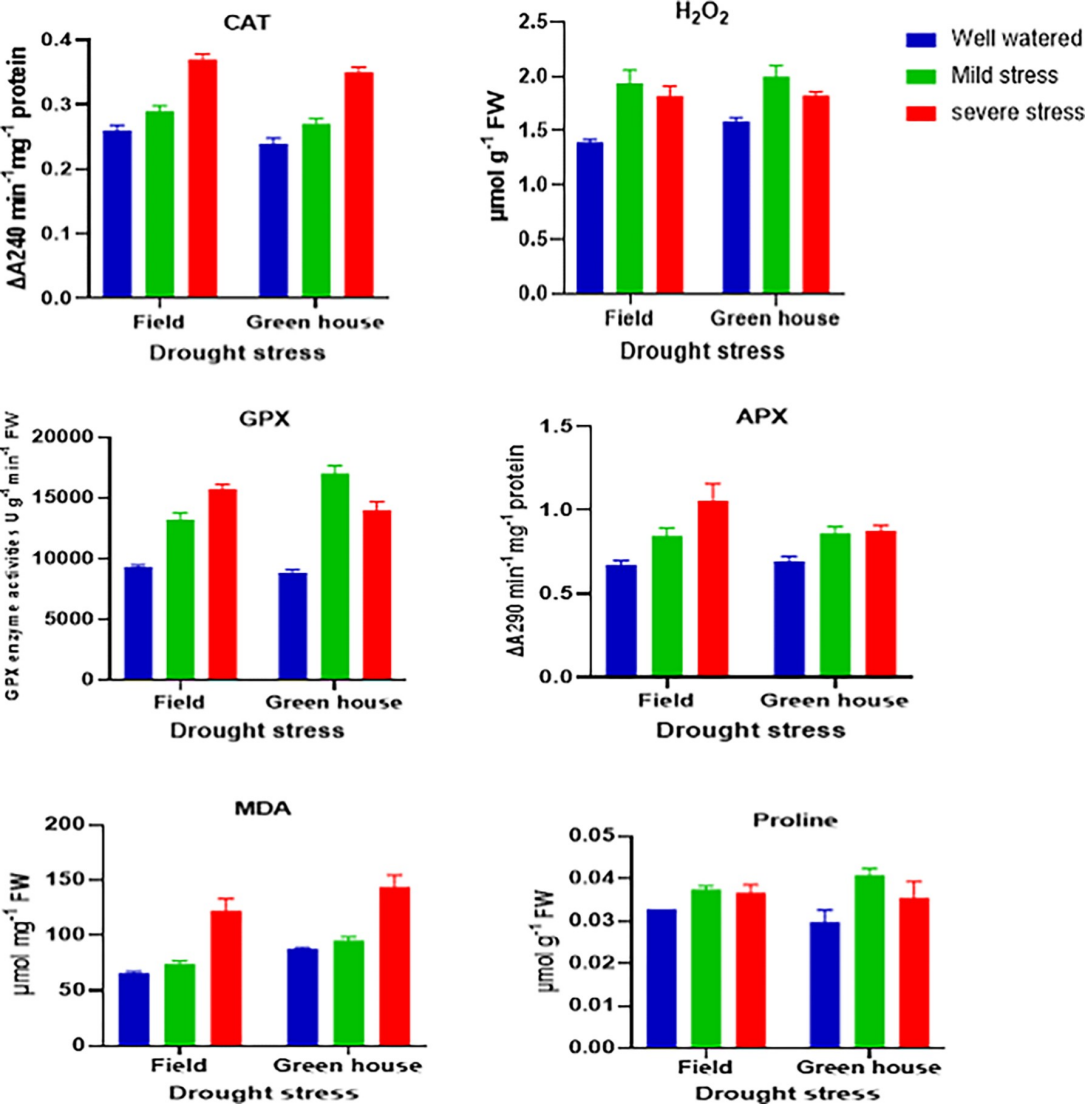
Catalase (CAT) activity, hydrogen peroxide (H_2_O_2_), guaiacol peroxidase (GPX), ascorbate peroxidase (APX), malondialdehyde (MDA) and proline content in leaves harvested from TKS plants grown under different levels of drought stress. Each value is the mean of 3 replicates ± SE.

**Fig 5:**
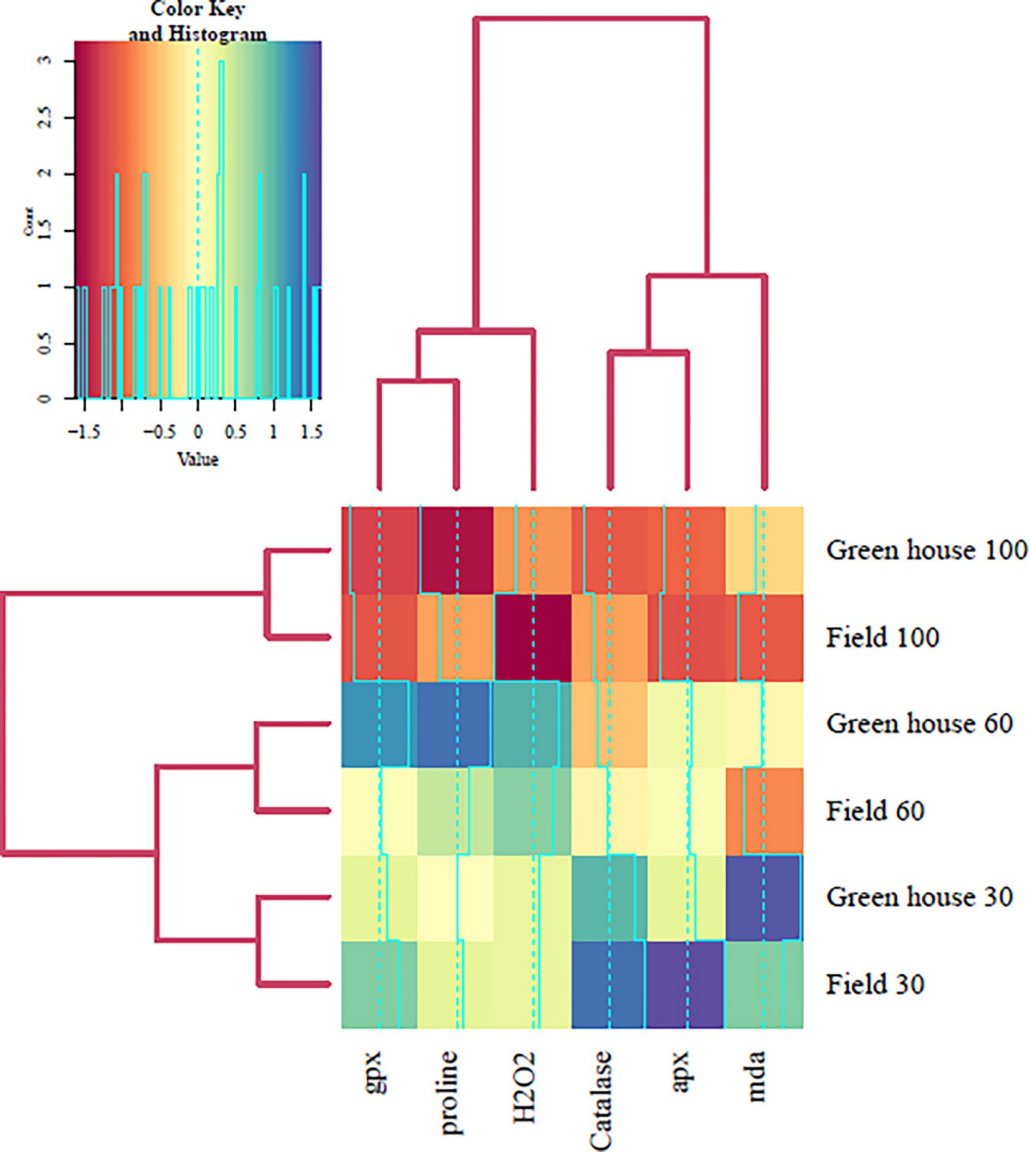
Shows the heatmap and cluster analysis of physiological parameters for both field and greenhouse conditions under different levels of drought stress. The levels of drought stress were 100 (well-watered), 60 (mild stress), and 30 (severe stress). The heatmap indicates the relative values of the physiological parameters, with darker colors representing higher values and lighter colors representing lower values. The cluster analysis shows the similarity of the physiological parameters and the drought stress treatments based on the Euclidean distance method. The dendrograms on the top and left sides of the heatmap represent the hierarchical clustering of the physiological parameters and the drought stress treatments, respectively.

Drought stress affected various physiological parameters of TKS leaves in both greenhouse and field conditions (Fig 4). The levels of malondialdehyde (MDA), hydrogen peroxide (H_2_O_2_), and free proline, which are indicators of oxidative stress and osmotic adjustment, increased significantly under water deficit. MDA, a product of lipid peroxidation caused by reactive oxygen species (ROS) such as hydroxyl radical (OH), superoxide ion (O2•–) and H_2_O_2_ [49], showed the highest accumulation under severe water deficit (30%) in both locations, with an increase of 64% in the greenhouse and 85% in the field compared to the control (p ≤ 0.01). H_2_O_2_ concentration also increased under drought stress, but the highest level was found under mild water stress (50%) rather than severe stress, with an increase of 26% in the greenhouse and 39% in the field compared to the control (p ≤ 0.01). Free proline, a compatible osmolyte that protects plants from dehydration and osmotic stress, accumulated under even mild water stress in both locations, with an increase of 37% in the greenhouse (p ≤ 0.01) and 14% in the field (p ≤ 0.05) compared to the control. The antioxidant enzyme activities of guaiacol peroxidase (GPX), catalase (CAT) and ascorbate peroxidase (APX) also increased significantly under drought stress in both locations (Fig 4). GPX activity reached the highest level under severe stress in the field and under mild stress in the greenhouse, with an increase of 69 and 93%, respectively, compared to the control (p ≤ 0.01). CAT and APX activities reached the highest levels under severe stress in the field, with an increase of 42% and 57%, respectively, compared to the control (p ≤ 0.01 and < 0.05, respectively). The effects of drought stress on physiological traits were analyzed by correlation and cluster analysis, as shown in Fig 5. The results indicated that drought stress increased the accumulation of physiological traits, and these traits had positive correlations with each other. Cluster analysis revealed that GPX and proline were highly correlated and belonged to the same cluster, followed by H_2_O_2_ in the next level of classification. In addition, CAT was correlated with APX, and then with MDA in the subsequent level. Moreover, the drought stress treatments for both locations were grouped in the same cluster.

### 3.2. Gene expression analysis

**Fig 6:**
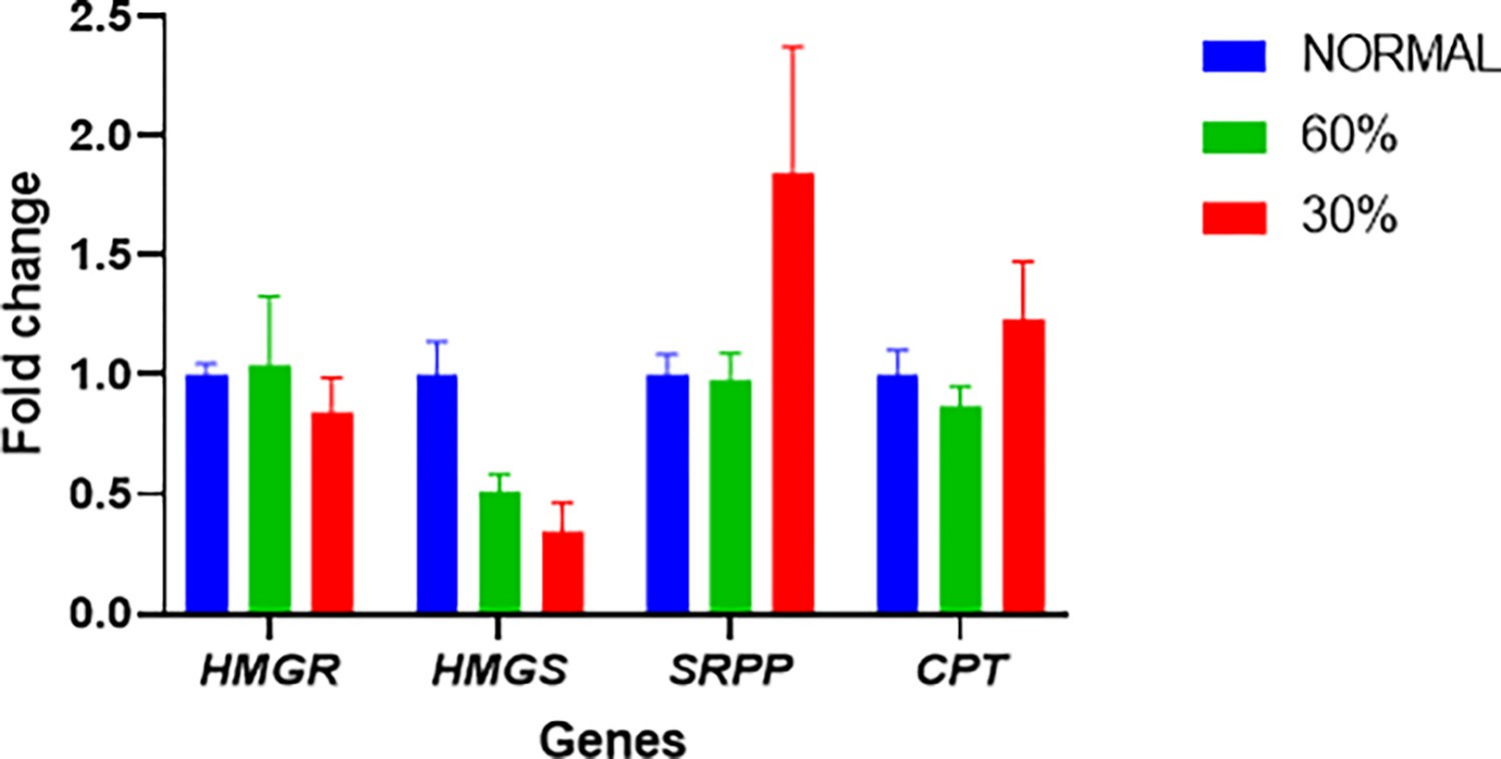
Relative expression of selected genes putatively involved in natural rubber biosynthesis in TKS root tips under different drought stress treatments. Each value is the mean of 3 replicates ± SE.

The expression levels of the selected genes under different drought treatments are shown in Fig 6. Drought stress had a significant effect on the expression of *HMGS1*, which was downregulated by fold change of 0.51 and with fold-change of 0.34 under moderate and severe drought, respectively, compared to the control. *SRPP4* was the only gene that was upregulated by drought stress, showing a 1.84-fold increase under severe drought. *CPT1* and *HMGR* showed minor changes in expression under drought stress, with *CPT1* increasing by 1.23-fold and *HMGR* decreasing by 0.84-fold under severe drought. The expression of the other genes wasn’t significantly affected by drought stress.

### 3.3. Rubber concentration

**Fig 7:**
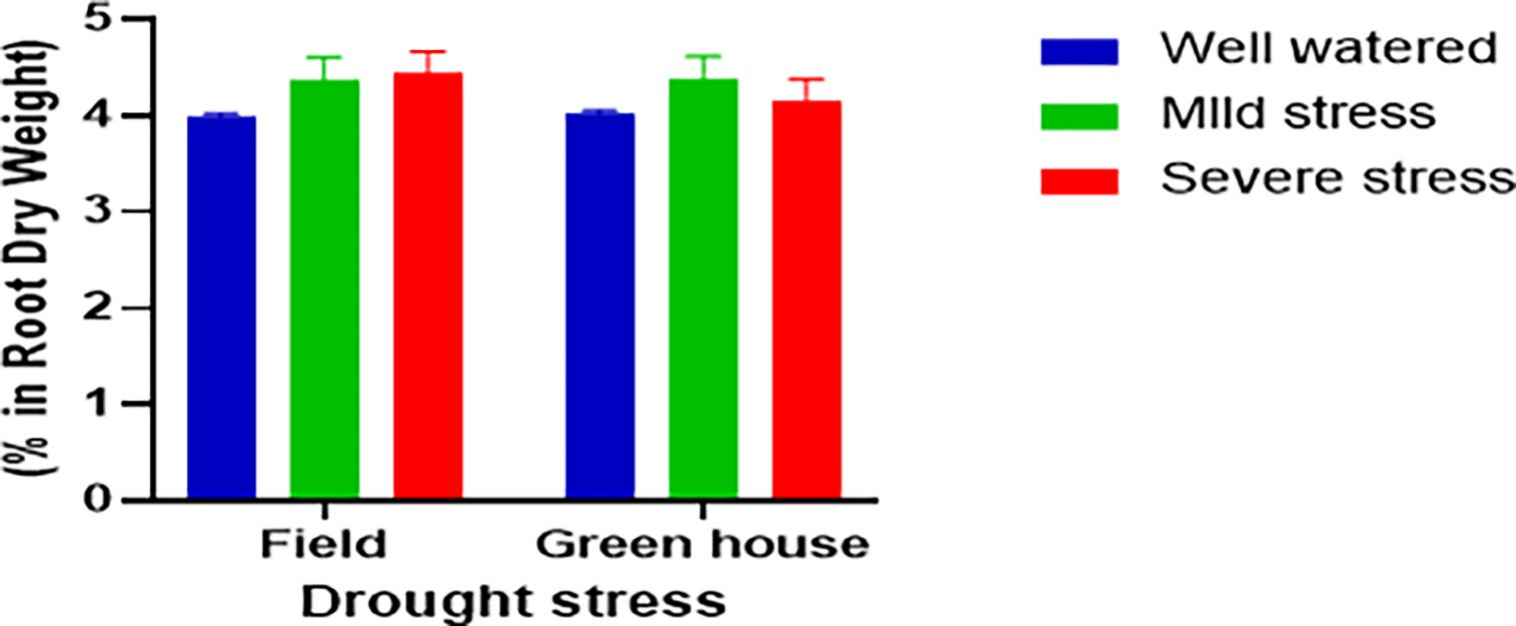
Rubber concentration in TKS roots. Each value is the mean of three replicates ± SE.

The rubber concentration in the TKS roots was measured under different levels of drought stress in both field and greenhouse conditions. The results indicated that there was no significant difference (p > 0.05) in the rubber content of roots among the treatments, although the roots under mild and severe drought stress had slightly higher rubber content (8 and 19%, respectively) than the control group (Fig 7). This indicated that drought stress didn’t have a significant effect on the rubber biosynthesis in the TKS roots. The expression of *HMGS*, a key enzyme for the production of the rubber monomer IPP, was also analyzed in the root tips under stress. The results showed that the *HMGS* expression was downregulated by 034-fold and 0.51-fold in the 30 and 60% drought stress groups, respectively, compared to the control (Fig 6). This suggests that the rubber biosynthesis pathway was inhibited by drought stress at the transcriptional level. The rubber content in TKS dry roots from the wild collection was consistent with our results, ranging from 4 to 4.5% (Fig 7).

### 3.4. Molecular weight distribution of TNR

The molecular weight (Mw) and polydispersity index (PDI) of the extracted TNR samples under different irrigation treatments are shown in Table 2. The Mw of TNR significantly increased under drought stress, while the PDI decreased. The molecular weight of TNR under normal irrigation was almost the same as the natural rubber from *H*. *brasiliensis* [50]. The PDI of TNR under normal irrigation was obtained 2.41, which indicated a broad molecular weight distribution. The Mw of TNR increased under drought stress, while the PDI decreased to 1.45. This suggested that drought stress may have induced the formation of longer polymer chains and reduced the chain branching of TNR.

**Table 2:** Molecular characteristics of TNR extracted from single samples from each greenhouse treatment.

Sample		Mw (g/mol)	Mn (g/mol)	Mp (g/mol)	PDI
Control (90%)		0.99*10^6^	4.12*10^5^	1.04*10^6^	**2.41**
Mild stress (60%)		1.31*10^6^	9.08*10^5^	1.17*10^6^	**1.45**
Severe stress (30%)		1.12*10^6^	6.30*10^5^	1.04*10^6^	**1.77**

### 3.5. Differential scanning calorimetry (DSC)

**Fig 8:**
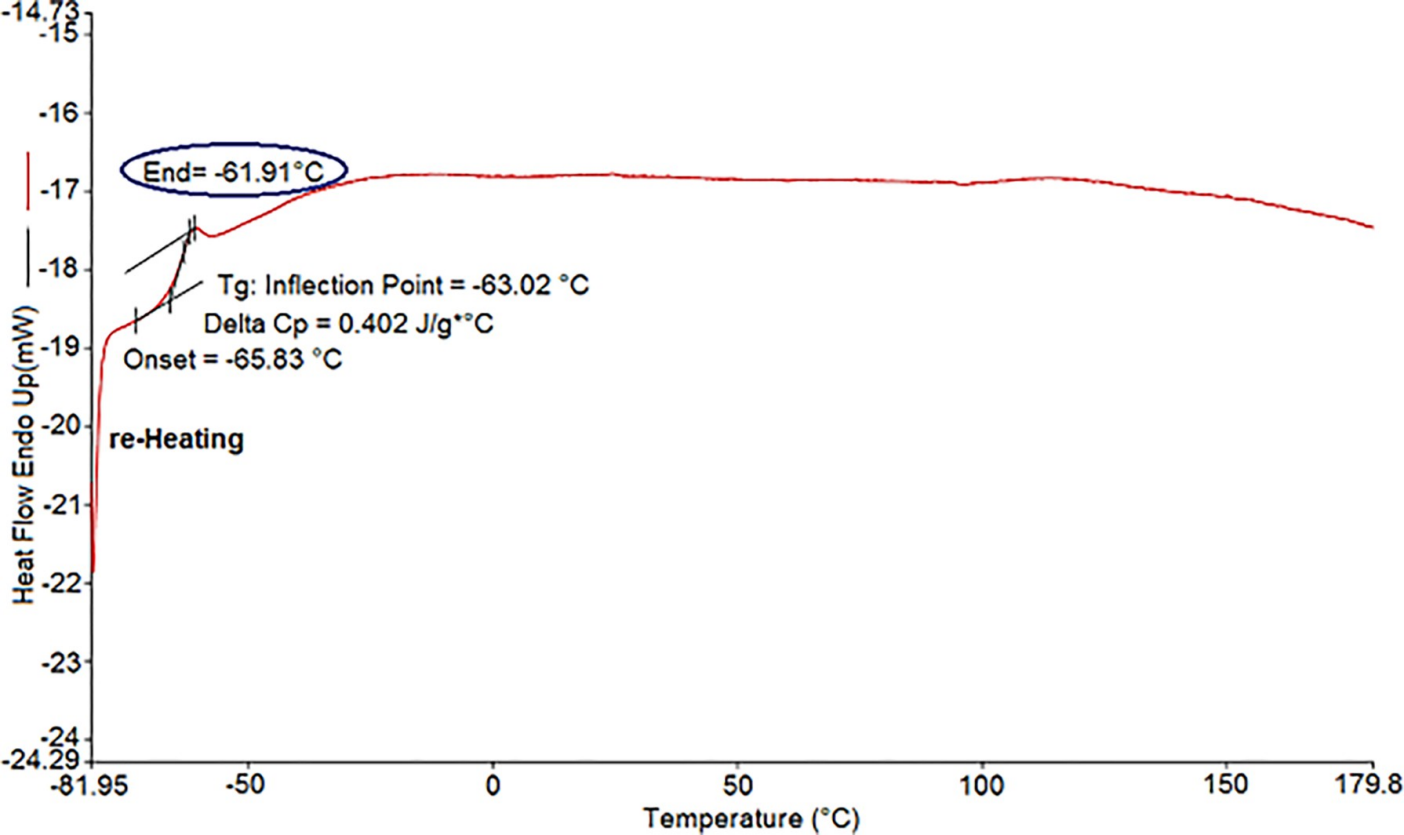
Differential scanning calorimetry (DSC) analysis of NR.

TNR’s glass transition temperature (Tg) was measured by DSC and shown in Fig 8. The Tg value was -63.02°C and the heat capacity at the Tg was 0.402 J/g*°C.

### 3.6. Fourier Transform Infrared Spectroscopy (FTIR)

**Fig 9:**
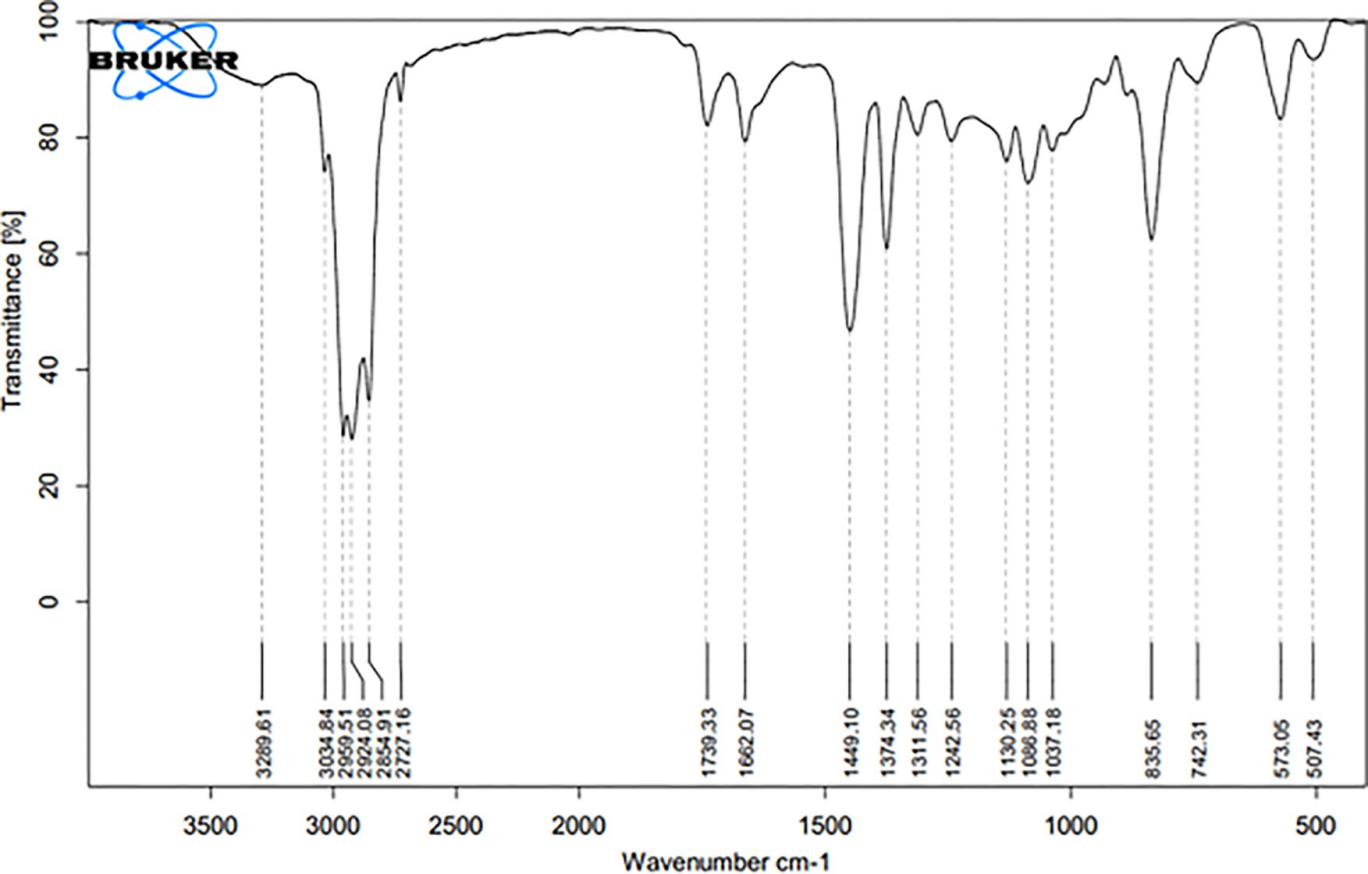
FTIR analysis of TNR.

FTIR confirmed that the TNR sample was *cis*-1,4, polyisoprene (Fig 9). The main peaks observed below 1,000 are the unique peaks for *cis*-1,4 bonds [44, 51] wave number 835 cm^-1^.

## 4. Discussion

The results indicated that drought stress triggered oxidative stress and osmotic adjustment in *TKS* leaves, as manifested by the elevated levels of MDA, H_2_O_2_, and free proline. These responses are consistent with those observed in other rubber-producing plants such as *H*. *brasiliensis* under water deficit conditions [52]. MDA is a product of lipid peroxidation and membrane damage caused by ROS, which are produced by various metabolic pathways under stress conditions [49]. The higher accumulation of MDA under severe water deficit implies that the antioxidant defense system was insufficient to cope with the excessive ROS generation. H_2_O_2_ is one of the main ROS that can act as a signaling molecule or a toxic agent depending on its concentration and location [53]. The highest level of H_2_O_2_ under mild water stress may suggest a higher activation of the respiratory burst oxidase system in the plasma membrane, which generates superoxide ions that are converted to H_2_O_2_ by SOD [54]. Alternatively, it may indicate a lower removal capacity of H_2_O_2_ by antioxidant enzymes such as GPX, CAT and APX under mild stress compared to severe stress. Free proline is a well-known compatible solute that accumulates in plants under various abiotic stresses such as drought, salinity, and low temperature [55, 56]. Proline accumulation can result from several factors, such as reduced protein synthesis, enhanced proline biosynthesis from glutamate or ornithine, decreased proline degradation or catabolism, and increased protein hydrolysis [55–57]. Proline can play multiple roles in plant stress tolerance, such as stabilizing membranes and proteins, scavenging ROS, maintaining cellular redox balance, regulating osmotic potential, and acting as a signaling molecule [58, 59]. Drought stress induced an increase in the antioxidant enzyme activities of GPX, CAT and APX in *TKS* leaves, suggesting a strengthened defense mechanism against ROS-induced oxidative damage. GPX reduces H_2_O_2_ to water using guaiacol as an electron donor, while CAT decomposes H_2_O_2_ to water and oxygen without requiring any cofactor. APX uses ascorbate as an electron donor to reduce H_2_O_2_ to water and regenerates ascorbate from monodehydroascorbate by MDHAR or DHAR [60]. These enzymes may have different roles and regulation in the antioxidant system under varying levels of drought stress [61, 62]. GPX may be more important for scavenging low levels of H_2_O_2_ under mild stress, whereas CAT and APX may be more effective for removing high levels of H_2_O_2_ under severe stress. The higher activity of GPX in the field than in the greenhouse under severe stress may also indicate a higher adaptation of *TKS* to natural conditions than to artificial conditions. A recent study by [63] showed that different drought-tolerant alfalfa varieties had different root morphological and physiological characteristics under PEG-induced drought stress. They found that the root diameter, root surface area, root length, and root–shoot ratio were significantly affected by drought stress, and that these traits were correlated with the levels of osmolytes, reactive oxygen species, and antioxidant enzymes and antioxidants in the root. In another study [64] reported that soybean plants subjected to the different levels of drought stress had reduced root surface area, root length, and root diameter, and increased xylem diameter in the root. They suggested that these anatomical changes were related to the physiological responses of the plant, such as CO2 assimilation, stomatal conductance, transpiration, and carboxylation efficiency. These studies show that drought stress affects the physiology and biochemistry of plant roots in significant ways, and that these effects may differ from those seen in the shoot or leaves. Another study found that drought stress increased the activity of antioxidant enzymes in both shoot and root tissues, implying that the antioxidant defense system plays a role in removing reactive oxygen species. The author also reported that watering the plants after drought stress partially restored their morphological and biochemical characteristics, indicating some degree of recovery from drought damage [65]. *SRPP4*, which belongs to a stress-related protein category [66], was overexpressed under severe drought. *SRPP* has been shown to stabilize rubber particles and increase rubber production in guayule under drought stress [67]. The co-localization of *CPT* with *SRPP* implies that they may also be upregulated by drought stress, as reported in *T*. *brevicorniculatum* [1, 18, 68, 69]. *CPTs* are involved in sterol and dolichol synthesis (a 15 to 23-mer of *cis*-polyisoprene) and cell membrane formation. Dolichol plays a role in drought stress tolerance in *Arabidopsis* [70] and in the antioxidant mechanisms of cell membranes, so drought stress may increase *CPT* gene expression through the enhancement of antioxidant enzyme activities [71]. This work revealed new aspects of the molecular mechanisms underlying rubber biosynthesis and drought stress response in *TKS*. The expression levels of key genes involved in rubber biosynthesis, such as *HMGS1*, *SRPP4* and *CPT1*, were altered by drought stress, indicating that *TKS* may have a sophisticated regulation of rubber production under unfavorable environmental conditions. However, the increase in rubber concentration under drought stress could not be explained by enhanced rubber biosynthesis or reduced root biomass under stress, as the whole root weight data was inconclusive [72]. Moreover, the downregulation of *HMGS* suggests that the rubber biosynthesis pathway may be inhibited by drought stress in *TKS*. The rubber content in *TKS* dry roots from the wild collection may reflect the natural variation of rubber production among different genotypes or environmental conditions. The DSC results are in agreement with previous reports that showed similar Mw values for TNR, e.g., 1.4*106 g/mol [73, 74], suggesting that drought stress does not affect the polymerization of rubber monomers in *TKS*. The main finding of this paper is that drought stress does not decrease the total rubber content in *TKS* roots, but rather improves the quality of the extracted rubber for industrial applications. This may be attributed to the increased stability of rubber particles under stress, as evidenced by the higher expression of *SRPP4* (Fig 5). The Tg of TNR was comparable to that of Hevea NR, which was reported to be -62.8°C by Jitsangiam et al. (2021) [75]. This indicates that the molecular mobility and chain flexibility of TNR were similar to those of Hevea NR, despite the different sources and extraction methods of the natural rubber [76]. The FTIR peak at 835 cm^-1^ corresponds to the *cis*-1,4 bond stretching vibration of polyisoprene [44, 51], confirming that the TNR sample was mainly composed of *cis*-1,4 polyisoprene, which is the characteristic structure of natural rubber. The other peaks at 1377, 1449, 1668 and 3070 cm^-1^ are related to the CH2 and CH3 groups of the isoprene units [51].

## 5. Conclusions

TKS was able to survive mild and severe stress in both greenhouse and field conditions without a significant loss in rubber properties. This suggests that TKS might be a suitable crop for semi-arid temperate regions or those regions that experience transient drought periods. Key metabolic indicators responded as expected as stress-related enzymes and genes changed in activity and expression, respectively. Further research is needed to determine the best agricultural practices, such as planting date, planting density and harvest date, and the commercial viability of TKS farming in different parts of Iran.
